# Million-atom molecular dynamics simulations reveal the interfacial interactions and assembly of plant PSII-LHCII supercomplex†

**DOI:** 10.1039/d2ra08240c

**Published:** 2023-02-27

**Authors:** Ruichao Mao, Han Zhang, Lihua Bie, Lu-Ning Liu, Jun Gao

**Affiliations:** a Hubei Key Laboratory of Agricultural Bioinformatics, College of Informatics, Huazhong Agricultural University Wuhan 430070 Hubei China gaojun@mail.hzau.edu.cn; b Institute of Systems, Molecular and Integrative Biology, University of Liverpool Liverpool L69 7ZB UK luning.liu@liverpool.ac.uk; c Frontiers Science Center for Deep Ocean Multispheres and Earth System, College of Marine Life Sciences, Ocean University of China Qingdao 266003 China

## Abstract

Protein–protein interface interactions dictate efficient excitation energy transfer from light-harvesting antennas to the photosystem II (PSII) core. In this work, we construct a 1.2 million atom-scale model of plant C_2_S_2_-type PSII-LHCII supercomplex and perform microsecond-scale molecular dynamics (MD) simulations to explore the interactions and assembly mechanisms of the sizeable PSII-LHCII supercomplex. We optimize the nonbonding interactions of the PSII-LHCII cryo-EM structure using microsecond-scale MD simulations. Binding free energy calculations with component decompositions reveal that hydrophobic interactions predominantly drive antenna–core association and the antenna–antenna interactions are relatively weak. Despite the positive electrostatic interaction energies, hydrogen bonds and salt bridges mainly provide directional or anchoring forces for interface binding. Analysis of the roles of small intrinsic subunits of PSII suggests that LHCII and CP26 first interact with small intrinsic subunits and then bind to the core proteins, whereas CP29 adopts a one-step binding process to the PSII core without the assistance of other factors. Our study provides insights into the molecular underpinnings of the self-organization and regulation of plant PSII-LHCII. It lays the framework for deciphering the general assembly principles of photosynthetic supercomplexes and possibly other macromolecular structures. The finding also has implications for repurposing photosynthetic systems to enhance photosynthesis.

## Abstract 2

Theoretical simulations reveal the general mechanism of antenna anchoring to the core in PSII.

## Graphical abstract

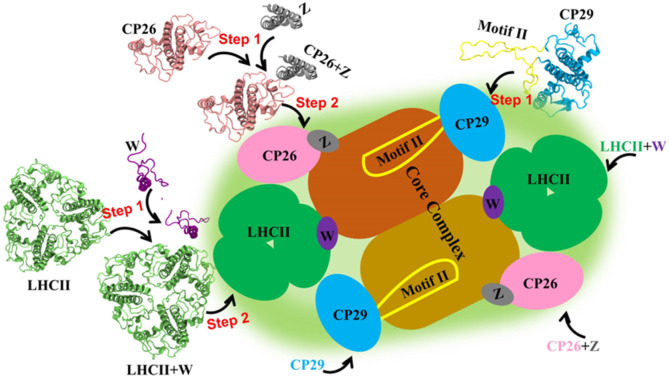

## Introduction

Photosynthesis is the primary source of energy for most life on Earth. It is estimated that photosynthesis produces more than 100 billion tons of dry biomass annually, which is equivalent to 100 times the weight of the current total human population on Earth and equal to an average energy storage rate of about 100 TW.^1^ The high-efficiency energy conversion is governed by the physiological coordination and arrangement of photosynthetically active chromoprotein complexes, which were generated during more than 3.5 billion years of evolution.^2^ Understanding the assembly and energy transfer mechanisms of photosynthetic protein complexes is pivotal for advancing our knowledge of the fundamentals of photosynthesis and underpinning the development of artificial photosynthesis to enhance and modulate energy conversion.^3^

Photosystem II (PSII) is the only enzyme that catalyzes water splitting in oxygenic photosynthesis and has been a primary target in designing biomimetic photosynthetic systems.^4^ Our knowledge about the assembly principles of the PSII core has been greatly improved by studies on high-resolution structures (up to 1.9 Å) and functions.^5–7^ Moreover, substantial developments have been made recently in PSII-based hybrid systems and the utilization of PSII assemblies for photocatalytic water splitting.^3,8,9^ For example, a hybrid natural-artificial photosynthetic platform was constructed by wiring a PSII core and a silicon photoelectrochemical cell, which could perform the water-splitting process under solar irradiation.^8^

Plant PSII dimeric core associates with membrane-spinning light-harvesting antenna complexes (LHCII) to form PSII-LHCII supercomplexes. The dynamic interactions between LHCII and PSII have been the major challenge in the high-resolution structural characterization of PSII-LHCII supercomplexes.^10^ This has been drastically improved by the technological advances of cryo-electron microscopy (cryo-EM), demonstrating the power in revolving large biomolecular assemblies.^11–18^ However, the resolved 3D structures reflect the static states of multiprotein supercomplexes under specific experimental conditions.^19^ They could not delineate dynamic protein associations and the assembly process of photosynthetic PSII-LHCII supercomplexes. Indeed, cryo-EM structures have suggested a higher degree of flexibility of atomic positions at the antenna-PSII core interface and dynamic associations of LHCII with the PSII core,^11^ which are fundamental for state transitions and PSII repair.^20–22^ Moreover, PSII-LHCII undertakes a stepwise assembly pathway, and the attachment of LHCII to the dimeric PSII core appears to occur at the final step of PSII-LHCII assembly.^23^ Some protein subunits, including PsbW, PsbZ, and PsbH, were suggested to be involved in the antenna-PSII core assembly.^11,23–26^ The detailed protein–protein interactions and assembly mechanisms that govern the association of LHCII with the PSII core remain elusive.

Molecular dynamics (MD) simulations provide a powerful toolkit to investigate *in silico* the dynamics of biomolecular assemblies with atomic resolutions at a time scale ranging from femtoseconds to milliseconds.^19,27^ Large-scale MD simulations have been applied in studying the structures and functions of photosynthetic macromolecular complexes and organelles at different scales.^17–19,28–30^ Here, we construct a million-atom scale model of plant C_2_S_2_-type PSII-LHCII supercomplex embedded in the solvated membrane, based on the cryo-EM structure,^11^ and perform microsecond-scale MD simulations to study the protein interactions and assembly process of the sizeable PSII-LHCII supercomplex in a near cellular environment. Our results indicate the general binding principles and atomistic details of the PSII-LHCII assembly.

## Methods

### Initial model

The cryo-EM structure of spinach PSII-LHCII (PDB ID: 3JCU)^11^ was selected as the initial model for simulating the interactions between LHCII proteins and the PSII core. According to the previous studies,^31–33^ the PSII-LHCII complex was embedded in a pre-equilibrated lipid bilayer consisting of single-component 1-palmitoyl-2-oleoyl-*sn-glycero*-3-phosphocholine (POPC) to mimics native thylakoid membranes. The system was then neutralized by the addition of counter ions (202 sodium ions) and solvated in a double-shell water box containing 277 727 TIP3P water molecules. Our simulation box (240.5 Å × 350.0 Å × 155.5 Å) contains a total of 1 159 431 atoms, and the solvated complexes with lipid bilayers are shown in Fig. S1.†

Histidine residues in the system were singly protonated on Nε, except those coordinating to non-heme FE, HEM or CLA *via* Nε (and were thus protonated on Nδ). In addition, the two disulfide bonds contained in each monomer (C112–C135 of PsbO and C17–C26 of PsbTn) were explicitly considered. The AMBER ff14SB force field parameter set^34^ was selected for standard amino acids residues. For the ten cofactors (PHO, BCR, PL9, LHG, SQD, LMG, DGD, LUT, XAT, NEX) (full names are shown in Table S1†), the generalized Amber force field (GAFF) parameter set was adopted.^35^ The parameters of CLA and CHL were taken from those developed by Ceccarelli *et al.* for bacteriochlorophyll *a*.^36^ The parameters of HEM were taken from the AMBER parameter database.^37^ The parameters of POPC were taken from previous work.^38^ For the non-heme Fe clusters, we obtained the parameters using the MCPB.py program.^39^ The atomic charges of the cofactors were determined by fitting the electrostatic potential around these molecules by using the RESP model.^40^ The atomic charges of the OEX complexes were assigned according to the redox states of its atoms in the dark-adapted (S1) state as follows: Mn1–Mn2, +3; Mn3–Mn4, +4; O1–O5, −2, Ca, +2. According to Ogata *et al.*,^41^ the equilibrium bond lengths, bond angles, and torsion angles were set as the average value of OEX in the two monomers in the cryo-EM structure. The force constants for bond lengths, bond angles, and torsion angles were appropriately set at 1500 kcal mol^−1^ Å^−2^, 500 kcal mol^−1^ Å^−2^, and 100 kcal mol^−1^ Å^−2^, respectively, to maintain the configuration of OEX during the simulations.

### Molecular dynamic simulations

Energy minimization of the whole system was performed with a three-step procedure: (1) the system was minimized with 20 000 steps with the steepest descent algorithm by freezing the PSII-LHCII complex to smooth the contact between the phospholipid membrane and the protein. (2) 10 000 steps of energy minimization were performed for the whole system with restraints on the protein backbone and heavy atoms of cofactors (100 kcal mol^−1^ Å^−2^) (3) 10 000 steps of energy minimization was performed without any restraints. The whole system was then slowly heated to 300 K within 60 ps under the restraint (10 kcal mol^−1^ Å^−2^) to the protein backbone and heavy atoms of cofactors. After these optimization and heating procedures, the constraints were released, and 10 ns equilibrium simulations were performed under the *NPT* ensemble at 300 K to balance the dimensions and density of the system. Finally, a 1 μs production MD simulation was performed using the GPU implementation of PMEMD from the AMBER16 software package.^42^ Atomic coordinates of all atoms were recorded every 1 ps. Temperature is controlled here using the Langevin thermostat^43^ while pressure is controlled using the anisotropic Berendsen barostat.^44^ Covalent bonds involving hydrogen atoms were constrained using the SHAKE algorithm.^45^ A non-bonding cutoff of 10 Å was applied to van der Waals interactions, and the particle mesh Ewald (PME) method^46^ was used to deal with long-range electrostatics.

### Binding free energy analysis and identification of hot spots

The structure and function of proteins are extremely sensitive to the surrounding environment. For biomolecular in aqueous environments, a general molecular mechanics Poisson–Boltzmann surface area (MM/PBSA) approach is used, which replaced the explicit solvent with an implicit continuous solvent. However, for membrane proteins, the effect of the membrane environment must be properly considered to ensure accuracy. In this work, the implicit membrane model developed by Luo *et al.*^47^ was used to examine the binding free energy between the antennas and the PSII core complex. Unfortunately, the implicit membrane model does not support energy decomposition. So, the classical implicit solvent model approach was also applied.

The MM/PBSA method calculates the binding free energy of the complex (Δ*G*_bind_) by eqn (1). In eqn (1), −*T*ΔS is the contribution of entropy to the system's free energy, which mainly involves the energy changes caused by conformational changes such as translation and rotation of the protein. Given that there is no significant change in protein subunits conformation, and the analysis mainly focuses on the electrostatic and hydrophobic contributions of the system rather than the absolute binding free energy, the contribution of the entropy (−*T*ΔS) was not calculated as that in recent literature.^48–50^ Δ*G*_gas_ is the sum of the electrostatic interaction energy Δ*G*_elec_ and the van der Waals interaction energy Δ*G*_vdW_ in vacuum (eqn (2)), representing the contribution of the molecular potential energy. The solvation energy includes the electrostatic solvation energy Δ*G*_PB_ and the non-polar solvation energy Δ*G*_np_ (eqn (3)).

1 Δ*G*_bind_ = Δ*G*_gas_ + Δ*G*_sol_ − *T*ΔS

2 Δ*G*_gas_ = Δ*G*_elec_ + Δ*G*_vdW_

3 Δ*G*_sol_ = Δ*G*_PB_ + Δ*G*_np_

4 *G*_np_ = *γ* × SASA + *b*

For the solvated PSII-LHCII complexes with lipid bilayers, the binding free energy of the protein–protein interfaces was calculated using the MM/PBSA approach with the MMPBSA.py module in AmberTools20. For the implicit membrane model, the heterogeneous dielectric membrane model is used here because it describes the membrane environment more accurately than the single dielectric membrane model.^47^ The spline fitting was adopted, and the implicit membrane thickness was obtained by calculating the average explicit membrane thickness of the last 800 ns trajectory. This was accomplished by calculating the location of the center of mass of the N and P atoms in the phosphatidylcholine headgroups at the top of the membrane, performing the same calculation on the bottom of the membrane, and taking the difference between them. Periodic boundary conditions were used here so that the value of the electrostatic solvation energy Δ*G*_PB_ is always 0, as it is part of the vacuum electrostatic interaction energy Δ*G*_elec_. Other PB settings are consistent with Greene *et al.*^47^ The internal dielectric constant settings for protein in the implicit membrane model and the implicit solvent model will be discussed in the next section. Δ*G*_np_ is calculated by eqn (4), where the values of constant *γ* and constant *b* are set to 0.005 kcal mol^−1^ Å^−2^ and 0.92 kcal mol^−1^, respectively. Solvent accessible surface area (SASA) was calculated using a water probe radius of 1.4 Å.

The free energy contributions of protein residues could be divided into polar (Δ*G*_polar_) and nonpolar (Δ*G*_nonpolar_) interactions according to eqn (5) where each part is the sum of two energy terms, as shown in eqn (6) and (7). In the following analysis, Δ*G*_polar_ is considered as the contribution of electrostatic interactions, while Δ*G*_nonpolar_ is considered as the contribution of hydrophobic interactions.^48,49^

5 Δ*G*_bind_ = Δ*G*_polar_ + Δ*G*_nonpolar_

6 Δ*G*_polar_ = Δ*G*_elec_ + Δ*G*_PB_

7 Δ*G*_nonpolar_ = Δ*G*_vdW_ + Δ*G*_np_

According to the previous literature,^51^ the hot spots are identified as those whose absolute free energy value is larger than 1.5 kcal mol^−1^. 8000 snapshots were extracted at intervals of 100 ps along the trajectory. It should be mentioned that since the PSII-LHCII complex is a dimer, the binding free energy values are discussed using the average of the two monomers.

## Results

### Construction of the structural model of PSII-LHCII for MD simulations

**Fig. 1:**
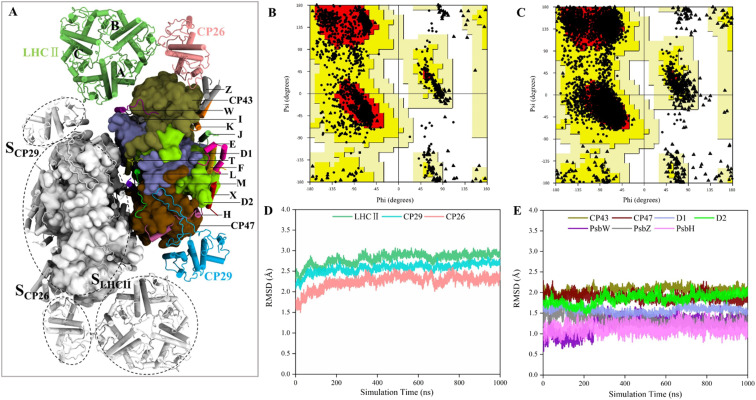
Validation of the quality of MD simulation. (A) Stromal view on the PSII-LHCII dimer. For clarity, only the subunits of the right monomer are highlighted, and different subunits are colored and labeled differently (the four extrinsic subunits are not shown). Among them, the 4 large core subunits are shown using the surface model and the remaining parts are shown using the cartoon model. The positions of three antennae–core interfaces are indicated in the left monomer. (B and C) Ramachandran plots for cryo-EM and MD structures, respectively. (D and E) RMSD values of proteins associated with the antennae–core interfaces (for CP26 and PsbW, only the RMSDs of non-loop regions were considered due to the high volatility of the loop regions). The RMSDs are obtained using the average of the two monomers and are colored as in (A).

The structures of several PSII-LHCII complexes from different species have been solved.^12–14,52^ In this work, the cryo-EM structure from spinach PSII-LHCII (PDB ID: 3JCU) was selected as the initial model for simulating the interactions between antenna proteins and the PSII core.^11^ As shown in Fig. 1A, the spinach C_2_S_2_-type PSII-LHCII supercomplex forms a homodimer with two-fold symmetry. Each monomer comprises a PSII core, a major peripheral light-harvesting complex (LHCII), and two minor chlorophyll-binding proteins of 26 and 29 kDa (CP26, CP29). The PSII core contains four large intrinsic subunits (D1, D2, CP43 and CP47), four extrinsic subunits (PsbO, PsbP, PsbQ, PsbTn) on the luminal surface (Fig. S1†), and twelve small membrane-spanning subunits (PsbE, PsbF, PsbH, PsbI, PsbJ, PsbK, PsbL, PsbM, PsbTc, PsbW, PsbX and PsbZ). Three light-harvesting complexes are bound to the core complex through the LHCII–core, CP26–core, and CP29–core interfaces, which display different protein contacts. Here, we define the three interfaces as *S*_LHCII/core_, *S*_CP26/core_, and *S*_CP29/core_, respectively. In addition, LHCII also forms interfaces with CP26 and CP29, defined as *S*_LHCII/CP26_ and *S*_LHCII/CP29_, respectively. This PSII-LHCII structure was embedded in a pre-equilibrated lipid bilayer consisting of single-component 1-palmitoyl-2-oleoyl-*sn-glycero*-3-phosphocholine (POPC) to mimic native thylakoid membranes (Fig. S1†).^31–33^ The model construction and the MD simulation process of the PSII-LHCII complex are shown in ESI Video 1.†

The conformations from the 1 μs MD production run were compared to the cryo-EM structure to check the quality of MD simulations. The average structure was obtained over a 1 μs trajectory, and the snapshot with the smallest RMSD value from the average structure was adopted as the representative structure. We found that the representative structure has only slight differences with the cryo-EM structure (Fig. S2†). First, Ramachandran plot analysis from PROCHECK^53^ revealed that the MD structure has a wider conformational distribution space and most of the backbone in the allowed regions of Ramachandran plot, compared with the cryo-EM structure (Fig. 1B and C). The number of residues in disallowed regions was reduced from 0.6% to 0.2%, and the number in generously allowed regions was reduced from 0.6% to 0.3% (Table 1). The number of residues in the most favored regions was similar (88.1% for MD structure and 88.3% for cryo-EM structure). These results confirmed the good quality of the cryo-EM structure and indicated that MD simulations only slightly improved the stereochemistry of the cryo-EM structure. Then, we examined the quality of nonbonded interactions using ERRAT program.^54^ The structures were evaluated based on the quality factor, which depends on the statistics of nonbonded atomic interactions in the 3-D protein structure. The results showed that the overall quality factor of the MD structure (90.2%) was greater than that of the cryo-EM structure (79.8%) (Table 1), indicating that MD simulations did improve the quality of nonbonded interactions from the PSII-LHCII cryo-EM structure. Since the antenna–core binding mainly depends on nonbonded interactions between the protein interfaces, the improvement in the accuracy of nonbonded interactions here is very important for the subsequent accurate calculation of protein interface affinities.

**Table 1:** Validation of cryo-EM and MD structures using PROCHECK and ERRAT program

Structure	Ramachandran plot statistics (%)				ERRAT (%)
	Most favored	Additionally allowed	Generously allowed	Disallowed	Overall quality factor
Cryo-EM	88.3	10.5	0.6	0.6	79.8
MD	88.1	11.4	0.3	0.2	90.2

### Conformational fluctuation analysis

To verify whether the antenna–core interfaces reach equilibrium, we calculated the RMSD values of three antennas (LHCII, CP29, CP26) and seven PSII core subunits (4 large intrinsic subunits D1, D2, Cp43, Cp47, and 3 small intrinsic subunits PsbW, PsbZ, PsbH). As shown in Fig. 1D and E, after 200 ns of simulation, the RMSD values of all the proteins at the antenna–core interfaces remained consistent, indicating that the *S*_LHCII/core_, *S*_CP26/core_, and *S*_CP29/core_ interfaces reached stable states. Likewise, RMSD analysis showed that the pigments also reached equilibrium (Fig. S3†). Accordingly, the MD simulation trajectories after 200 ns were used for subsequent statistical analysis.

**Fig. 2:**
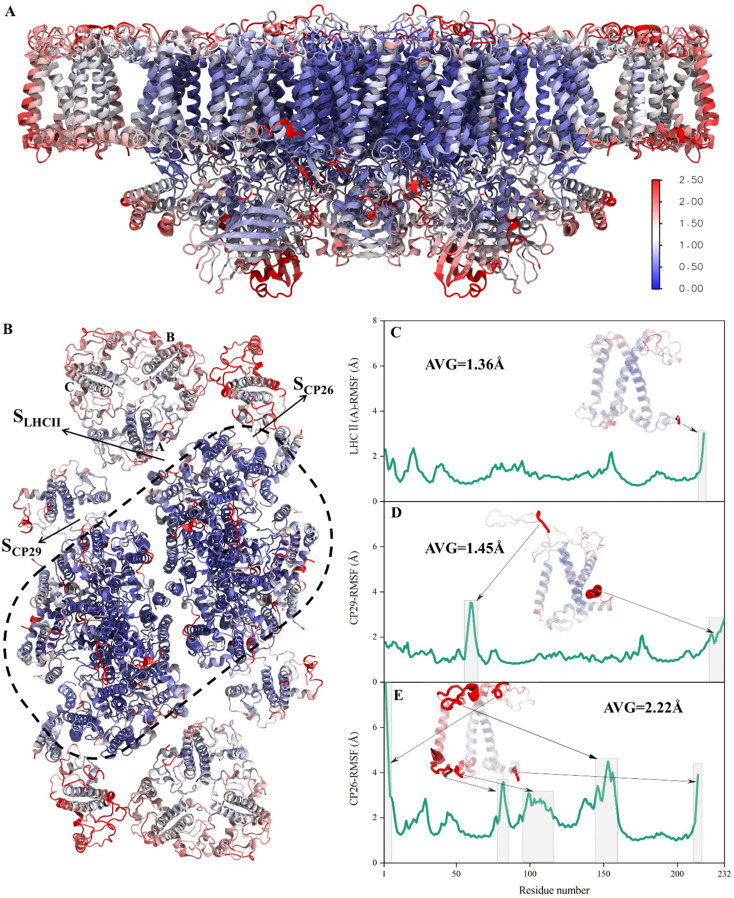
RMSF fluctuations of the PSII-LHCII backbone. (A) Side view and (B) stromal view on the PSII-LHCII dimer. All protein residues were colored according to the RMSF values of C_α_ atoms capped at 2.5 Å: red represents a high RMSF value and blue represents a small RMSF value. (C–E) RMSF values of LHCII(A), CP29 and CP26 residues, respectively. Grey boxes and arrows indicate the regions with higher fluctuation values.

The RMSD values of the peripheral antennas LHCII, CP29, and CP26 (ranging from 1.50 Å to 3.07 Å, Fig. 1D) were generally more significant than those of the core proteins (ranging from 0.58 Å to 2.31 Å, Fig. 1E), suggesting that the light-harvesting antennas possess larger conformational fluctuations than the PSII core subunits, consistent with the experimentally observed high mobility of antenna proteins.^10^ To further characterize the fluctuation features of protein residues, we investigated root mean square fluctuation (RMSF) of the protein backbone C_α_ atoms around their average positions. The volatility of extrinsic subunits (average RMSF value of 1.91 Å, Fig. 2A) and antenna proteins (average RMSF value of 1.71 Å, Fig. 2B) was markedly higher than that of the core complex (average RMSF value of 1.16 Å, Fig. 2B), consistent with RMSD results (Fig. 1). In addition, the fluctuation of pigments was strongly correlated with the fluctuation of binding proteins (Fig. S4†). For individual antennas, CP26 has a notably higher RMSF value (average value: 2.22 Å) than LHCII monomer adjacent to the core (LHCII(A), average value: 1.36 Å) and CP29 (average value: 1.45 Å). The greater fluctuation was mainly ascribed to the loop regions of CP26, which is away from the *S*_CP26/core_ interface; likewise, the fluctuating regions of CP29 and LHCII(A) are also not at the antenna–core interfaces (Fig. 2C–E). Consequently, although the peripheral antennas showed overall conformational fluctuations, *S*_LHCII/core_, *S*_CP26/core_, and *S*_CP29/core_ exhibited relatively stable conformations (Fig. 2B), presumably favoring the association of antennas with the PSII core and energy transfer. The analysis also verified the reliability of MD simulations using the constructed structural model.

### Analysis of the interface binding free energies

To study the binding characteristics at different antenna–core interfaces, the binding free energies of the three antenna–core interfaces (*S*_LHCII/core_, *S*_CP26/core_, and *S*_CP29/core_) were calculated using the MM/PBSA approach.^48,51,55^ As the choice of internal dielectric constant values has a significant impact on the results,^56^ we examined a set of values using *S*_LHCII/core_ as an example (Table S2†) to determine an appropriate internal dielectric constant. For the implicit membrane model,^47,57^ a value of 20 is appropriate when the ligand is charged, according to recommended settings in the literature.^57^ Therefore, we tested values around 20, including 10, 15, 20, 25, and 30. The change in binding free energy was examined with internal dielectric constant values of 1–8, often used for the implicit solvent model.^58,59^ The results showed that both models' free energy decreased with the internal dielectric constant increased, and only the electrostatic interaction energy changed (Table S2†). In addition, the electrostatic interaction energy was always positive, whereas the contribution of hydrophobic interactions remained negative. These results suggest that the choice of internal dielectric constants values does not have remarkable effects on qualitative determination of binding affinity. Therefore, the common setting, namely the internal dielectric constant value of 20 for the implicit membrane model and 1 for the implicit solvent model, was used to investigate the binding free energy of protein interfaces.

Based on the heterogeneous implicit membrane model, free energies of the three antenna–core protein interfaces were obtained from equilibrium trajectories (*i.e.*, 200–1000 ns) (Table 2). To verify the convergence of the free energy calculations, we performed convergence tests for *S*_LHCII/core_ (−58.0 ± 7.2 kcal mol^−1^, −57.0 ± 7.9 kcal mol^−1^, −59.4 ± 7.2 kcal mol^−1^, −56.4 ± 13.6 kcal mol^−1^), *S*_CP26/core_ (2.3 ± 5.5 kcal mol^−1^, 1.5 ± 5.6 kcal mol^−1^, 2.0 ± 5.6 kcal mol^−1^, 3.2 ± 6.4 kcal mol^−1^) and *S*_CP29/core_ (−111.6 ± 9.6 kcal mol^−1^, −112.7 ± 9.7 kcal mol^−1^, −116.1 ± 9.4 kcal mol^−1^, −116.1 ± 9.8 kcal mol^−1^) using trajectories of 800–1000 ns, 600–1000 ns, 400–1000 ns and 200–1000 ns, respectively. In addition, non-equilibrium trajectories (0–50 ns) were also used to calculate the binding free energies of *S*_LHCII/core_ (−2.7 ± 14.8 kcal mol^−1^), *S*_CP26/core_ (12.2 ± 5.1 kcal mol^−1^) and *S*_CP29/core_ (−84.5 ± 8.7 kcal mol^−1^), the results are clearly different from the above calculations using equilibrium trajectories, indicating that a long simulation scale is necessary for accurate free energy calculations of the antenna–core interface.

**Table 2:** Binding free energy of different antenna–core interfaces (kcal mol^−1^). Δ*G*_vdW_, the van der Waals interaction energy; Δ*G*_np_, the non-polar solvation energy; Δ*G*_elec_, the electrostatic interaction energy; Δ*G*_nonpolar_, the contribution of hydrophobic interactions; Δ*G*_polar_, the contribution of electrostatic interactions; Δ*G*_bind_, the binding free energy

Interface	Δ*G*_vdW_	Δ*G*_np_	Δ*G*_elec_^a^	Δ*G*_nonpolar_^b^	Δ*G*_polar_^c^	Δ*G*_bind_^d^
*S*_LHCII/core_	−103.5 ± 12.5	−11.4 ± 1.0	58.5 ± 2.5	−114.9	58.5	−56.4 ± 13.6
*S*_CP26/core_	−46.5 ± 5.3	−6.2 ± 0.5	55.9 ± 3.0	−52.7	55.9	3.2 ± 6.4
*S*_CP29/core_	−167.4 ± 8.5	−18.5 ± 0.7	69.8 ± 3.8	−185.9	69.8	−116.1 ± 9.8

a Includes the electrostatic solvation energy, see methods.

b Formula (6).

c Formula (7).

d Formula (5).

*S*_CP29/core_ (−116.1 kcal mol^−1^) has the largest total free energy among the three interfaces, which is double of *S*_LHCII/core_ (−56.4 kcal mol^−1^), indicating the strong binding affinity at *S*_CP29/core_. The electrostatic interaction energies (Δ*G*_elec_) of *S*_LHCII/core_, *S*_CP26/core_, and *S*_CP29/core_ were 58.5 kcal mol^−1^, 55.9 kcal mol^−1^ and 69.8 kcal mol^−1^. In contrast, their hydrophobic interaction energies (Δ*G*_np_) were −114.9 kcal mol^−1^, −52.7 kcal mol^−1^, and −185.9 kcal mol^−1^, respectively. As described in Methods (eqn (6) and (7)), the binding free energies can be divided into Δ*G*_polar_ and Δ*G*_nonpolar_ (hydrophobic interaction). Our analysis suggested that Δ*G*_nonpolar_ provides the dominant contribution to the binding of the antenna to the PSII core. Meanwhile, the Δ*G*_polar_ values of all interfaces are positive, indicating that polar or electrostatic interactions between the antenna and PSII provide repulsive forces unfavorable for binding. This conclusion was also supported by the calculation using the implicit water model (Table S3†). In contrast, the binding free energies of the two antenna–antenna interfaces, *S*_LHCII/CP26_ and *S*_LHCII/CP29,_ were both positive values (Table S4†), indicative of the very weak antenna–antenna association (Table 2).

### Free energy decomposition and hot spot analysis

Based on the identification of hydrogen bonds (HBs) and salt bridges (SBs) at the antenna–core interface in the cryo-EM structure,^11^ electrostatic interactions were previously assumed to be the main driving force mediating the binding of antennae to the PSII core.^60^ However, our free energy calculation indicated that the total electrostatic interactions act as repulsive forces. To address this contradiction, we examined the contributions of individual residues to the total free energies and HB/SB interactions formed by the interface residues. The free energy contributions were decomposed into polar and non-polar fractions to evaluate the contributions of electrostatic and hydrophobic interactions. Residues with an absolute value of binding free energy greater than 1.5 kcal mol^−1^ were identified as hot spots (Tables S5 and S6†), according to previous work.^51^

**Fig. 3:**
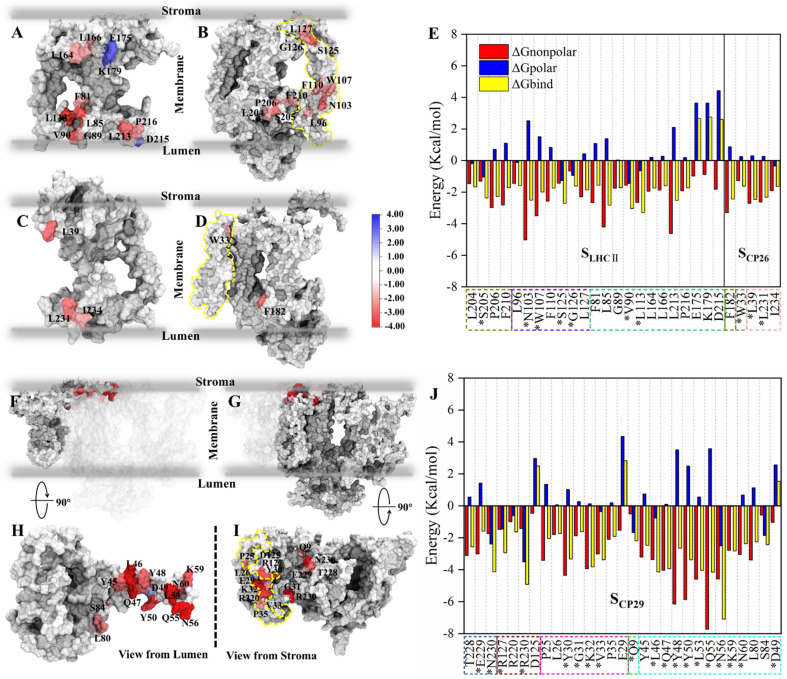
Binding free energy and corresponding component contribution of hot spots on *S*_LHCII/core_, *S*_CP26/core_ and *S*_CP29/core_ and their localization. (A and B) Localization of hot spots on LHCII and the core complex (at the *S*_LHCII/core_), respectively. (C and D) Localization of hot spots on CP26 and the core complex (at the *S*_CP26/core_), respectively. The protein surface map colored according to the free energy contribution of each hot spot. The yellow dotted lines in panels (B) and (D) correspond to the positions of the small intrinsic subunits PsbH and PsbZ, respectively. The unit of energy is kcal mol^−1^. (E) Binding free energy and corresponding component contribution of hot spots, including hydrophobic (red), electrostatic (blue) and total (yellow) contribution. Color scheme is presented as the same as shown in Fig. 1A. The dotted box lines with different colors represent different subunits, which are CP43 (tan), PsbW (purple), LHCII (lime), PsbZ (silver) and CP26 (pink). Hot spots that participate in the formation of hydrogen bonds or salt bridges are marked with *. See Table S5† for more details. (F and H) Localization of CP29 and corresponding hot spots. (G and I) Are the localization of core complex (at the *S*_CP29/core_) and corresponding hot spots. The yellow dotted lines in panel (i) correspond to the positions of the small intrinsic subunits PsbH. (J) Binding free energy and corresponding component contribution of hot spots. The dotted box lines with different colors represent different subunits, which are CP47 (ochre), D1 (ice blue), PsbH (mauve), PsbL (green), and CP29 (cyan), and the colors correspond to Fig. 1A. See Table S6† for more details.

For *S*_LHCII/core_, 23 amino acids were identified as hot spots (Fig. 3E and Table S5†). Among them, 20 hot spots had negative free energy values, and their total free energy contribution was −42.22 kcal mol^−1^, accounting for 65% of the total binding free energies (−64.5 kcal mol^−1^). Three residues in LHCII, E175, K179, and D215, have positive free energy contributions. All Δ*G*_nonpolar_ of residues are negative, but Δ*G*_polar_ of 13 residues are positive. For *S*_CP26/core_, only 5 amino acids were identified as hot spots (Fig. 3E and Table S5†), consistent with the relatively low interface affinity (Table 2). Interestingly, Δ*G*_polar_ of most of the residues are positive (Fig. 3E and Table S5†). A large number of hot spots at *S*_CP29/core_ (Fig. 3J and Table S6†), compared with those at *S*_LHCII/core_ and *S*_CP26/core_, is consistent with its higher interface affinity (Table 2). 26 of the 29 hot spots contribute negative free energies. Their total free energy contribution was −79.54 kcal mol^−1^, accounting for 68% of the total binding free energy. In contrast, 19 hot spots have positive Δ*G*_polar_ contribution. The free energy decomposition indicated that the Δ*G*_polar_ contribution (*i.e.* electrostatic interactions) is primarily positive, representing unfavorable interactions, consistent with our free energy analysis (Table 2). It should be noted that the residues with hydrogen bonds may contribute negative Δ*G*_polar_; we will discuss it in the next section.

We also determined the spatial distribution of hot spots. At *S*_LHCII/core_, 12 hot spots are located in LHCII, including 4 hot spots (L164, L166, E175, K179) at the stromal side and 8 hot spots (F81, L113, L85, V90, G89, L213, P216, D215) at the luminal side. Eleven hot spots are located in the core (Fig. 3A and B), including 3 hot spots (L127, G126, and S125) at the stromal side and 8 near the luminal side. At *S*_CP26/core_, 3 hot spots are located at CP26, and 1 hot spot is in the core complex, all of which are near the luminal side or the stromal side (Fig. 3C and D). At *S*_CP29/core_, all hot spots are concentrated at the stromal side: 16 hot spots were localized in the core complex, and 13 were localized at CP29 (Fig. 3F–I). Moreover, the hot spots in the core complex are widely distributed in multiple protein chains: 7 of the 16 hot spots are located at PsbH, 4 hot spots are at CP47, 3 hot spots are at D1, and the remaining one is localized at PsbL (Fig. 3I). At CP29, all the 13 hot spots are localized at Motif II (Pro42–Phe87) of the N-terminal long loop chain, implying its role of CP29–core complex binding. Collectively, the hot spots are mainly distributed at the luminal or stromal side, indicating that the main driving force in these interfaces' binding originates from the luminal or stromal side.

### Hydrogen bonds/salt bridges across the antenna–core interfaces

To further determine the effects of electrostatic interactions on the interfacial affinity, HBs/SBs at the antenna–core interfaces were analyzed. The distance for determining the formation of HBs was set to 3.5 Å, and the corresponding angle was 135°.^32,61^ SB analysis was performed using a homemade python script, and a cutoff distance of 6 Å was used to detect SBs between the basic nitrogen and acidic oxygens.^62^ HBs/SBs with an occupancy greater than 30% during MD simulations were considered stable. In addition to the HBs/SBs characterized in the cryo-EM structure (Table S7†), 25 additional HB/SB interactions were identified between the antenna–core interfaces during the course of 1 μs MD simulations at room temperature (Tables 3 and 4). Intriguingly, many of these HBs/SBs have been reported in other PSII-LHCII structures at higher resolutions,^13,52^ confirming the reliability of the structural model achieved from MD simulations.

Hydrogen bonds and salt bridges at *S*_LHCII/core_ and *S*_CP26/core_

**Table:** 

	Hydrogen bond		Distance (Å)	Occupancy (%)	PSII subunits	Cryo-EM structures^a^
	Donor	Acceptor				
*S*_LHCII/core_	VAL_90@N	ASN_103@Oδ	2.96 ± 0.16	64	PsbW	
	ASN_88@Nδ	PRO_97@O	2.88 ± 0.14	38	PsbW	
	ASN_103@Nδ	LEU_113@O	2.92 ± 0.15	95	PsbW	
	SER_205@N	ALA_214@O	2.92 ± 0.15	66	CP43	
	ASN_103@Nδ	VAL_90@O	2.94 ± 0.15	88	PsbW	
	GLY_126@N	GLU_175@Oε	3.03 ± 0.19	88	PsbW	
	TRP_107@Nε	TYR_112@Oη	3.14 ± 0.17	32	PsbW	
	SER_101@N	ASN_88@O	2.99 ± 0.16	37	PsbW	
	SER_125@Oγ	GLU_175@Oε	2.64 ± 0.11	34	PsbW	
*S*_CP26/core_	ARG_32@Nη	SER_143@O	2.90 ± 0.17	37	CP43	
	TRP_33@Nε	GLY_38@O	2.92 ± 0.16	61	PsbZ	5XNL, 7OUI
	SER_59@Oγ	LEU_231@O	2.78 ± 0.17	31	PsbZ	3JCU, 5XNL
	LYS_37@Nζ	LEU_39@O	2.88 ± 0.15	40	PsbZ	3JCU, 5XNL

a Interfacial HBs/SBs present in the cryo-EM structure of spinach (PDB ID: 3JCU, 3.2 Å), pea (PDB ID: 5XNL, 2.7 Å) and arabidopsis (PDB ID: 7OUI, 2.79 Å).

**Table:** 

	Salt bridge		Distance (Å)	Occupancy (%)	PSII subunits	Cryo-EM structures^a^
	Acidic	Basic				
*S*_CP26/core_	ASP_41	LYS_37	3.99 ± 0.92	47	PsbZ	5XNL
	ASP_44	LYS_37	5.05 ± 1.00	42	PsbZ	5XNL

Hydrogen bonds and salt bridges at *S*_CP29/core_

**Table:** 

Hydrogen bond		Distance (Å)	Occupancy (%)	Proteins in PSII	Cryo-EM structures^a^
Donor	Acceptor				
TYR_48@N	GLY_31@O	2.94 ± 0.14	99	PsbH	3JCU, 5XNL
ASN_56@Nδ	GLU_8@O	2.92 ± 0.15	96	PsbL	
GLN_47@Nε	TYR_30@O	2.92 ± 0.16	81	PsbH	3JCU
GLN_55@N	ASN_7@Oδ	2.99 ± 0.18	81	PsbL	
LEU_46@N	VAL_33@O	2.94 ± 0.14	87	PsbH	5XNL, 7OUI
TYR_50@Oη	SER_132@Oγ	2.85 ± 0.16	59	CP47	
ASN_56@Nδ	ASN_5@O	2.84 ± 0.12	56	PsbL	
ASN_56@N	PRO_6@O	2.98 ± 0.16	48	PsbL	
GLN_55@Nε	THR_10@O	3.03 ± 0.19	34	CP47	3JCU, 5XNL, 7OUI
ASN_60@Nδ	ARG_476@O	2.90 ± 0.14	50	CP47	5XNL
GLN_55@Nε	ASN_230@Oδ	2.97 ± 0.17	45	D1	5XNL, 7OUI
ASN_56@Nδ	PRO_6@O	2.92 ± 0.16	33	PsbL	
SER_84@Oγ	GLU_29@Oε	2.68 ± 0.13	32	PsbH	
VAL_33@N	LEU_46@O	2.91 ± 0.14	99	PsbH	7OUI
ASN_230@Nδ	ASN_56@Oδ	2.90 ± 0.15	95	D1	5XNL, 7OUI
GLU_229@N	LEU_57@O	3.02 ± 0.17	83	D1	5XNL
GLN_9@Nε	ASN_56@Oδ	2.90 ± 0.14	88	PsbL	5XNL
ASN_230@N	GLN_55@O	3.03 ± 0.16	59	D1	3JCU, 7OUI
GLN_223@Nε	TYR_48@Oη	3.07 ± 0.18	48	CP47	
ASN_14@Nδ	LEU_53@O	2.97 ± 0.17	40	CP47	7OUI
ARG_230@Nη	ASN_60@Oδ	2.94 ± 0.16	77	CP47	
ARG_230@Nη	ASP_51@Oδ	2.80 ± 0.11	48	CP47	
LYS_137@Nζ	GLN_81@Oε	2.84 ± 0.13	86	CP47	
ARG_476@Nη	LEU_53@O	2.98 ± 0.18	30	CP47	3JCU
ARG_230@Nε	ASP_51@Oδ	3.08 ± 0.18	30	CP47	

a Interfacial HBs/SBs also present in the cryo-EM structure of spinach (PDB ID: 3JCU, 3.2 Å), pea (PDB ID: 5XNL, 2.7 Å) and arabidopsis (PDB ID: 7OUI, 2.79 Å).

**Table:** 

Salt bridge		Distance (Å)	Occupancy (%)	Proteins in PSII	Cryo-EM structures^a^
Acidic	Basic				
GLU_85	LYS_32	3.80 ± 0.45	95	PsbH	3JCU, 7OUI
ASP_49	LYS_130	5.34 ± 0.39	55	CP47	3JCU
GLU_71	ARG_127	4.32 ± 0.93	43	CP47	
ASP_51	ARG_230	4.47 ± 0.29	71	CP47	
ASP_477	LYS_59	5.35 ± 0.33	39	CP47	3JCU, 7OUI
ASP_483	LYS_59	4.07 ± 1.23	79	CP47	3JCU, 5XNL

**Fig. 4:**
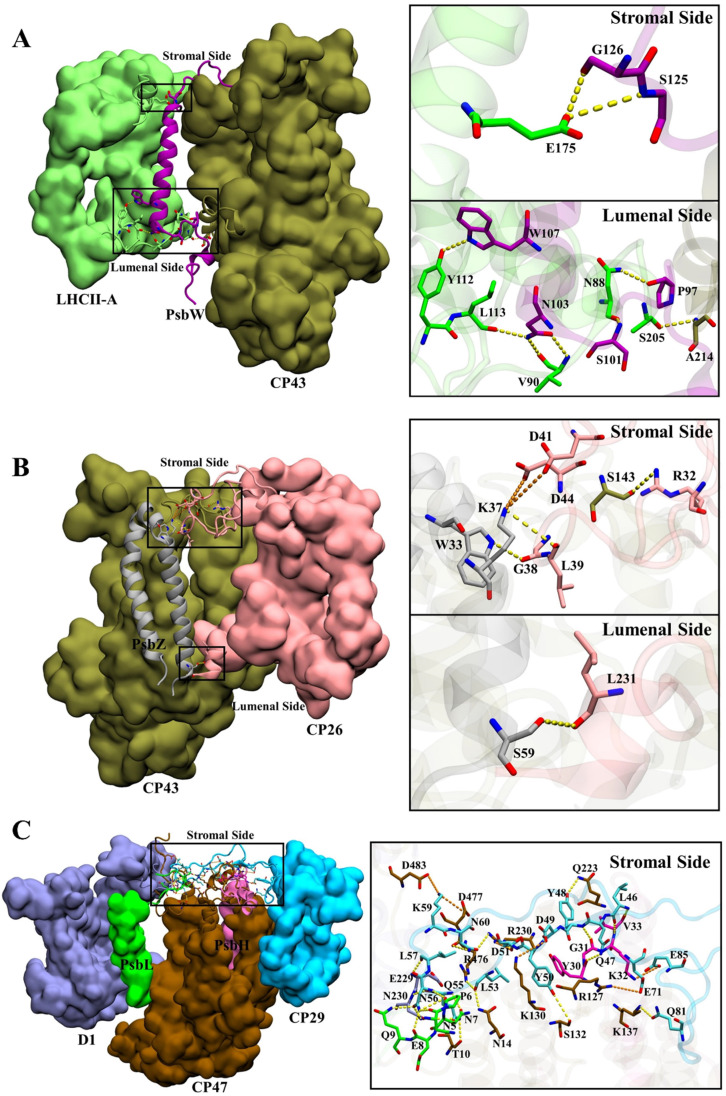
Hydrogen bond and salt bridge interactions at *S*_LHCII/core_ (a), *S*_CP26/core_ (b), and *S*_CP29/core_ (c). Hydrogen bonds and salt bridges are represented by yellow and orange dashed lines, respectively. Colour scheme is presented as the same as shown in Fig. 1A.

The total numbers and spatial locations of HBs/SBs found at *S*_LHCII/core_, *S*_CP26/core_, and *S*_CP29/core_ are consistent with those of the hot spots identified. The numbers of HBs/SBs are 25/6 for *S*_CP29/core_, 9/0 for *S*_LHCII/core_, and 4/2 for *S*_CP26/core_ (Table 3). Eight of the 9 HBs at *S*_LHCII/core_ are mediated by PsbW, and 7 are distributed at the luminal side (Fig. 4A and Table 3). Three of the 4 HBs and all SBs at *S*_CP26/core_ are mediated by PsbZ, and 3 HBs/SBs are located at the stromal side (Fig. 4B and Table 3). All the 25 HBs and 6 SBs at *S*_CP29/core_ are distributed at the stromal side and are mediated by Motif II of CP29. Residues of Motif II form HBs/SBs with multiple protein subunits of the PSII core (PsbH: 5/1; PsbL: 6/0; CP47: 10/5; D1: 4/0) (Fig. 4C and Table 4).

The differences between average binding free energies of the HB/SB-involved hot spots and all hot spots were −0.38 kcal mol^−1^ for *S*_LHCII/core_, −0.04 kcal mol^−1^ for *S*_CP26/core_, and −0.66 kcal mol^−1^ for *S*_CP29/core_ (Fig. S5†), which accounted for 26%, 1.6%, and 27% of the average binding free energies of all hot spots, respectively. This suggests that the formation of HBs/SBs predominately modulates hot spots' binding free energy contribution and enhances their binding ability. Moreover, the change in binding free energies was dominated by the reduction in electrostatic interaction energies (Fig. S5†). At *S*_LHCII/core_, 8 of the 23 hot spots were involved in HB formation (Fig. 3A–E, residues marked with *), and 5 hot spots, including V90 and L113 (LHCII), S125 and G126 (PsbW), and S205 (CP43), provide negative electrostatic interaction energies. At *S*_CP26/core_, 3 of the 5 hot spots (W33, L39, L231) were involved in HB formation and W33 contributes negative electrostatic interaction energies (Fig. 3C–E). At *S*_CP29/core_, 19 of the 29 hot spots were involved in HB/SB formation (Fig. 3J, residues marked with *), and 8 hot spots, including N230 (D1), R127 and R230 (CP47), V33 (PsbH), Q9 (PsbL), as well as L46, N56, and K59 (CP29), contribute negative electrostatic interaction energy. Overall, our results revealed that HBs/SBs are favorable for the binding of the interfaces, which is consistent to a certain extent with the previous study^60^ that indicated that electrostatic interactions were the main driving force to mediate the binding of antennae to the PSII core. However, our analysis showed that hydrogen bonds contribute only about 20% of the binding energies (Fig. S5†). In contrast, most of the binding energies come from Δ*G*_nonpolar_ (hydrophobic interaction). It is likely that hydrogen bonds mainly provide directional interactions and the anchoring of the interface.

### Roles of PSII small intrinsic subunits in antenna–core association

**Fig. 5:**
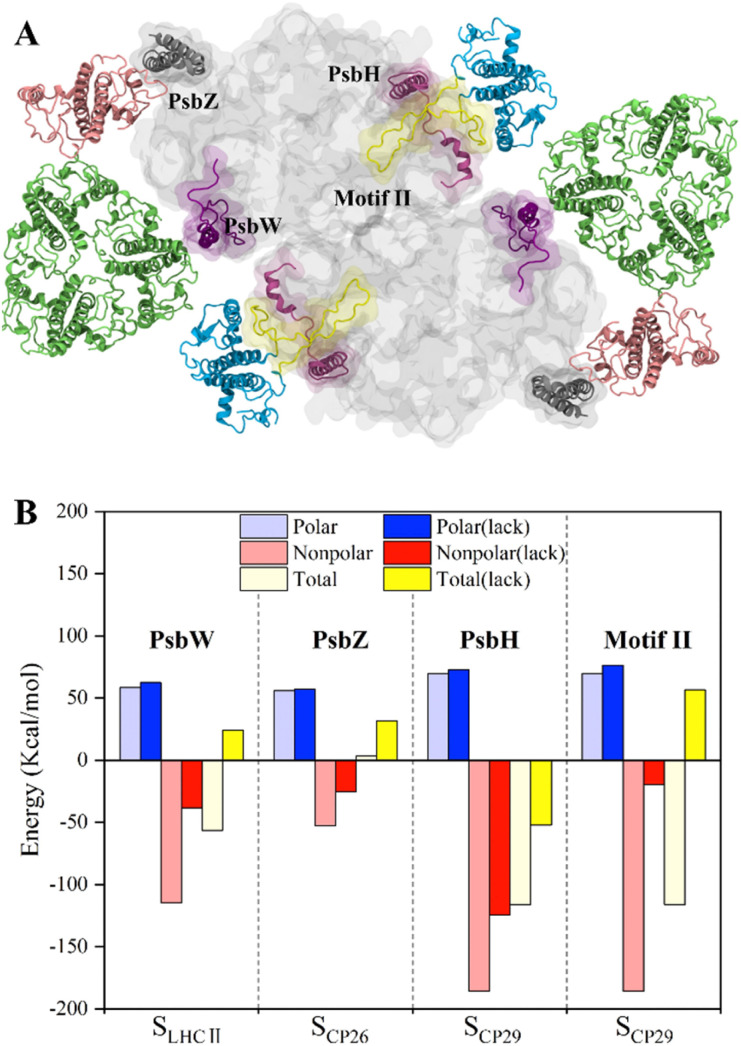
Binding free energy changes caused by the deletion of key subunits and a schematic diagram of the inferred assembly process. (A) Display of the small intrinsic subunits PsbW, PsbZ, PsbH, and Motif II of the N-terminal loop region of CP29, the colors of protein subunits correspond to Fig. 1a, and the Motif II is shown in yellow. (B) Affinity changes of the antenna–core interface in the absence of PsbW, PsbZ, PsbH and Motif II.

To study the roles of small intrinsic subunits in antenna–core binding, the binding free energies at the antenna–core interface in the absence of PsbW, PsbZ, and PsbH were calculated (Fig. 5A). The binding affinities at the antenna–core interfaces were significantly weakened in the absence of PsbW, PsbZ, and PsbH (Fig. 5B). The hydrophobic interaction energies of the ΔPsbW, ΔPsbZ, and ΔPsbH systems were decreased by 66.5%, 52.3%, and 32.9%, respectively, and the changes in electrostatic interaction energies were negligible. These results indicate that the small intrinsic subunits of PSII (PsbW, PsbZ, and PsbH) contribute about 30% to 60% of the binding free energies for antenna–core binding, corroborating the experimental findings that peripheral antenna content was reduced in the absence of PsbW and PsbZ.^24,25^

Most of the hot spots and residues involved in HB/SB formation of CP29 were identified on Motif II of CP29, which represents a long loop region (Pro42–Phe87) and is responsible for binding to the core proteins.^11^ In the absence of Motif II, the binding free energy increased from −116.1 ± 9.8 kcal mol^−1^ to 56.6 ± 4.1 (Fig. 5B), implicating that CP29 can not bind to core proteins without Motif II. Moreover, CP29 interacts with multiple subunits including PsbH, CP47, D1, and PsbL. This differs from LHCII and CP26, which interact with core proteins *via* small intrinsic subunits, *i.e.*, PsbW and PsbZ. The absence of PsbH only led to a relatively small percentage (32.9%) of binding free energy lose, suggesting that the function of PsbH in binding with CP29 and the core may not be as significant as expected.

## Discussion

High-resolution structures of PSII-LHCII supercomplexes have advanced our knowledge about the self-organization of photosynthetic antenna-reaction center systems. However, the relatively low resolution at highly dynamic regions in the systems, particularly the antenna–core interface, has raised many questions in understanding the PSII-LHCII assembly. Standing on the cryo-EM structure, we developed a reliable million-atom-scale model of plant C_2_S_2_-type PSII-LHCII supercomplex embedded in a solvated membrane using *in silico* simulations. Microsecond-scale MD simulations on the 1.1 megadalton multi-protein ensemble allow us to comprehensively analyze the inter-protein interactions and binding free energies at the antenna–core and antenna–antenna interfaces, providing very suggestive details of the stepwise assembly of the PSII-LHCII supercomplex.

In our study, the binding free energy calculations with corresponding component decompositions revealed that the antenna–core binding is a competitive process between electrostatic energy and hydrophobic energy, in which the electrostatic interactions provide mainly repulsive energy. In contrast, the hydrophobic contributions serve as the main driving force to facilitate antenna–core association (Fig. 3). This may imply a common mechanism underlying the protein–protein interactions and assembly of membrane macromolecular complexes, in which hydrophobic interactions play an essential role. In addition, compared to the cryo-EM structure, MD simulations reveal the HBs/SBs interactions at the antenna–core interface more comprehensively (Fig. 4). Electrostatic interaction energy analysis further indicated that the formation of HBs/SBs enhances the binding affinity of hot spots and is thought to play a positioning role during antenna–core assembly.

### Possible assembly pathways of PSII-LHCII, PSII-CP26 and PSII-CP29

**Fig. 6:**
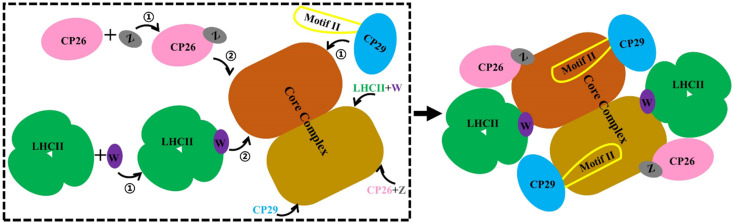
Schematic diagram of the antenna–core assembly process of the C_2_S_2_-type PSII-LHCII. LHCII is represented in green, CP26 in pink, CP29 in blue, PsbW in purple, and PsbZ in gray, these colors correspond to Fig. 1A. Motif II of CP29 is shown with a yellow coil. Brown and maroon show dimerized PSII cores. Arrows with numbers represent potential assembly steps.

Our MD simulations data supported a stepwise assembly of the PSII–LHCII complex (Fig. 6). The small intrinsic proteins PsbW and PsbZ bind to the periphery of the PSII core.^63^ They may play critical roles in mediating the binding of the antenna to the PSII core. Their absence, especially PsbW, could significantly weaken the hydrophobic interactions at the antenna–core interface (Fig. 5). The LHCII–core binding may undertake two steps: LHCII first binds to PsbW, due to its strong interaction with PsbW, to form the LHCII–PsbW assembly intermediate, and then binds to the PSII core as one assembly unit (Fig. 6). Previous experimental results supported that PsbW and LHCII co-occur with the PSII core.^23^ The CP26–core binding may also undertake two steps and form the CP26–PsbZ assembly intermediate before binding to the core. A previous experimental study has consistently shown that PsbZ could be released together with CP26 from the PSII complex.^23^ For CP29, *S*_CP29/core_ interaction analysis showed that binding to the PSII core was not strongly dependent on specific small intrinsic subunits (such as PsbH). Instead, CP29 forms extensive interactions with multiple core subunits of PSII through its Motif II to facilitate stable binding to the core. Therefore, it is more likely that the anchoring of CP29 to the PSII core represents a one-step process without the assistance of other factors and any assembly intermediate formation (Fig. 6).

### Weak interactions of the antenna–antenna interfaces support an independent assembly

Our data also indicated weaker binding affinity at the antenna–antenna interfaces than at the antenna–core interfaces, suggesting independent association/disassociation of individual antenna complexes LHCII, CP29, and CP26. Interestingly, under low-light conditions, two other antennas, M-LHCII and L-LHCII, could attach to the C_2_S_2_-type PSII-LHCII supercomplex.^12^ They do not interact directly with the PSII core but are bridged by CP29, ultimately forming the C_2_S_2_M_2_L_2_-type PSII-LHCII complex.^12^ These periphery antennae belong to the Lhcb protein family with similar sequence and structural features, suggesting that they may have similar interaction characteristics. It is known that LHCII can migrate between PSI and PSII to balance the distribution of excitation energy between PSI and PSII in state transitions.^20,21^ We speculate the interfacial affinities between M-LHCII/L-LHCII and CP29 are relatively weak, which might be essential for driving the dynamic association/dissociation of M-LHCII/L-LHCII to the PSII core in state transitions.^64,65^ Given the relatively strong interaction with the core, S-LHCII might be difficult for detach from the PSII core. This is consistent with previous experimental results, which showed that only L-LHCII freely diffuse between PSI and PSII in state transitions^66^ and that M-LHCII may detach from the PSII-LHCII supercomplex under specific physiological conditions.^65^ The detailed mechanisms underlying the dynamic PSII-LHCII assembly in state transitions remain to be elucidated. It has been presumed that phosphorylation of LHCII N-terminus sites could alter the binding affinity of LHCII to PSII, resulting in its disassociation from PSII.^20,66,67^ As electrostatic interactions provide repulsion between protein interfaces and protein residues at the stromal side of PSII-LHCII exhibits a negative electrostatic potential (Fig. S6†), phosphorylation of LHCII N-terminus likely leads to an increase in the negative electrostatic potential and the repulsive force at the protein–protein interfaces, which drive the disassociation of LHCII from PSII.

## Conclusions

Our structural model and simulations approach provide the framework for characterizing the molecular mechanisms of photosynthetic complexes' structural and functional dynamics. A better understanding of the antenna–photosynthetic reaction center assembly and interactions will inform strategies to enhance photosynthetic energy transfer and design efficient artificial photosynthetic systems.

## Author contributions

JG and LNL: idea conceptualization. JG and LNL: project administration, and funding acquisition. RM, LB, HZ, and JG: methodology and validation. RM, LB, JG and LNL: article writing.

## Conflicts of interest

There are no conflicts to declare.

## Supplementary Material

RA-013-D2RA08240C-s001

RA-013-D2RA08240C-s002
