# Supplementary material for: Million-atom molecular dynamics simulations reveal the interfacial interactions and assembly of plant PSII-LHCII supercomplex
Source: RSC Adv. 2023 Feb 27;13(10):6699–712. doi: 10.1039/d2ra08240c (PMC9969236; doi:10.1039/d2ra08240c)
Supplement: RA-013-D2RA08240C-s002 [file RA-013-D2RA08240C-s002.pdf]

Electronic Supplementary Material (ESI) for RSC Advances.
This journal is © The Royal Society of Chemistry 2023

Supplementary Information

for

Million-atom molecular dynamics simulations reveal the interfacial interactions and assembly of plant PSII−LHCII supercomplex"

Ruichao Mao[a], Han Zhang[a], Lihua Bie[a], Lu-Ning Liu[b,c,*], Jun Gao[a,*]

[a] Hubei Key Laboratory of Agricultural Bioinformatics, College of Informatics, Huazhong Agricultural University, Wuhan 430070 Hubei, China (Email: gaojun@mail.hzau.edu.cn)
[b] Institute of Systems, Molecular and Integrative Biology, University of Liverpool, Liverpool L69 7ZB, United Kingdom (Email: luning.liu@liverpool.ac.uk)
[c] Frontiers Science Center for Deep Ocean Multispheres and Earth System & College of Marine Life Sciences, Ocean University of China, Qingdao 266003, China

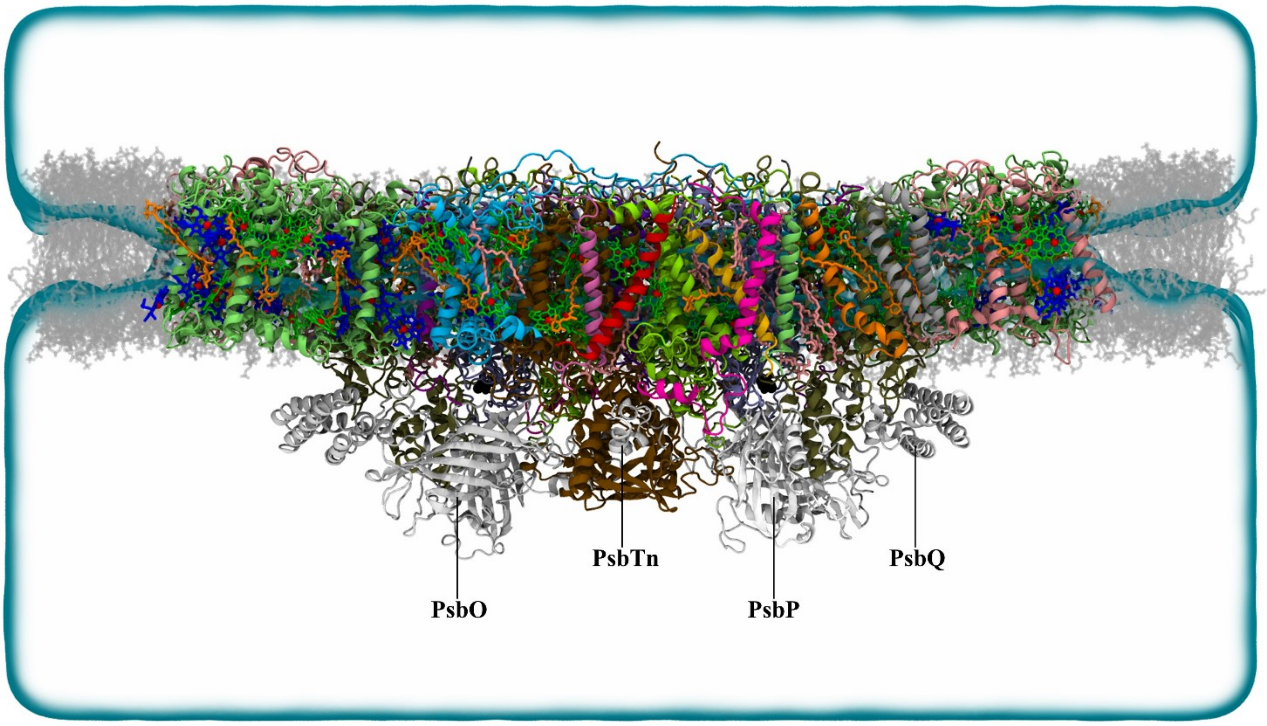

Fig. S1. Molecular dynamics model of solvated PSII-LHCII complex embedded in phospholipid membranes. The proteins are shown as cartoon models, in which the antenna and PSII core protein subunits are shown in different colors, and the four extrinsic subunits (PsbO, PsbP, PsbQ, and PsbTn) are shown in white. Cofactors are shown as licorice model, in which CLA is shown in green, CHL is shown in blue, carotenoids are shown in orange and other cofactors are consistently demonstrated in pink. The oxygen-evolving complex OEX is shown in black VDW.

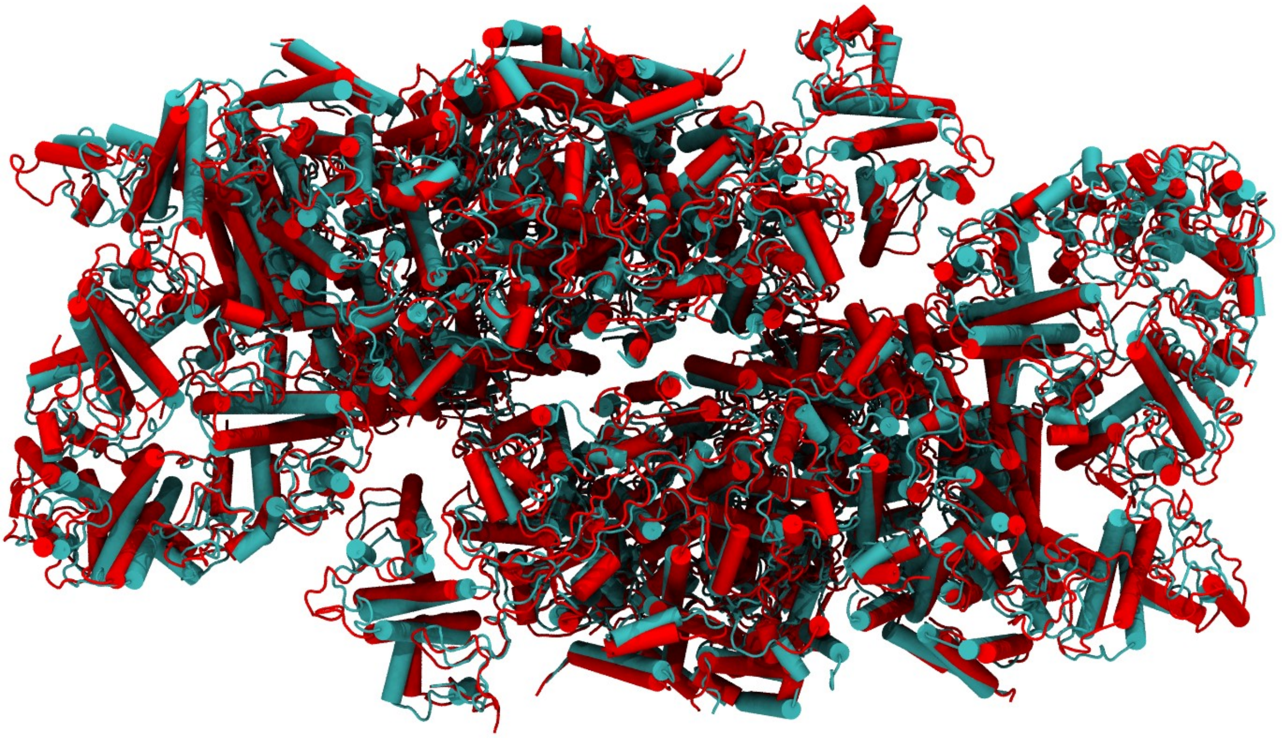

Fig. S2. Superposition of MD simulation structure and cryo-EM structure. The proteins are shown as cartoon models. Cyan represents the cryo-EM structure, and red represents the MD simulation structure.

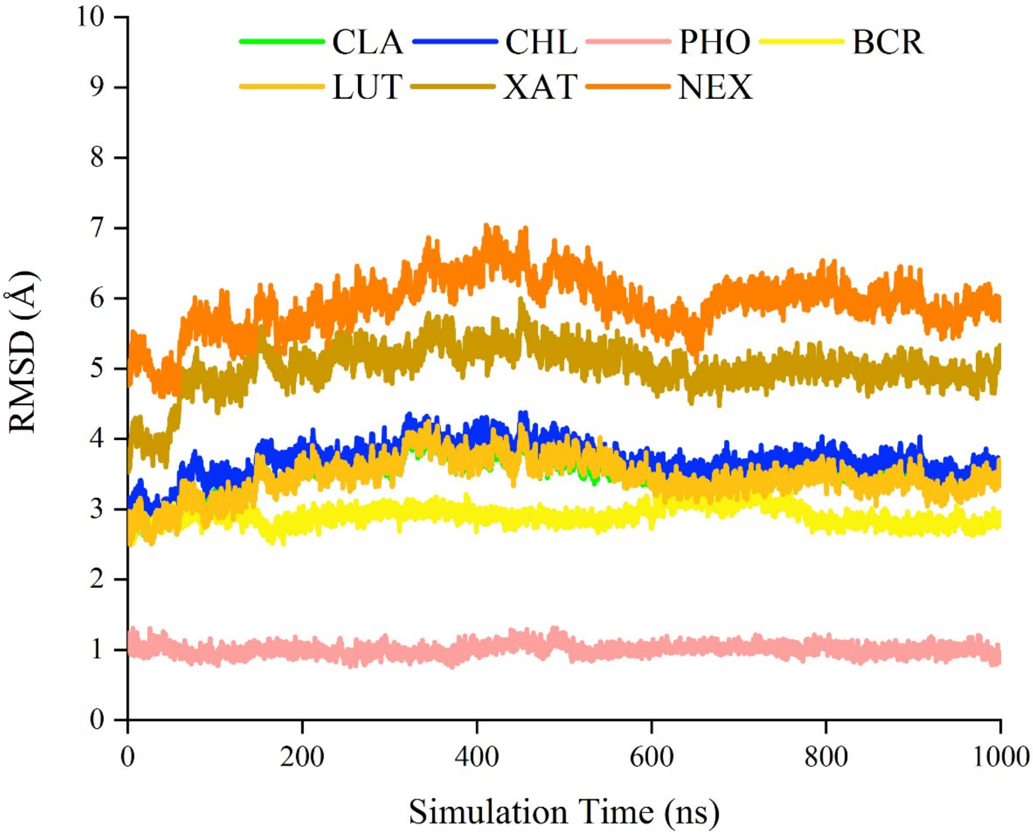

Fig. S3. RMSD of chlorophyll and carotenoid molecules. The colors of CLA (green), CHL (blue), PHO (pink), and the four carotenoids BCR, LUT, XAT, and NEX (shown as different depths of yellow) correspond to Supplementary Fig. 1.

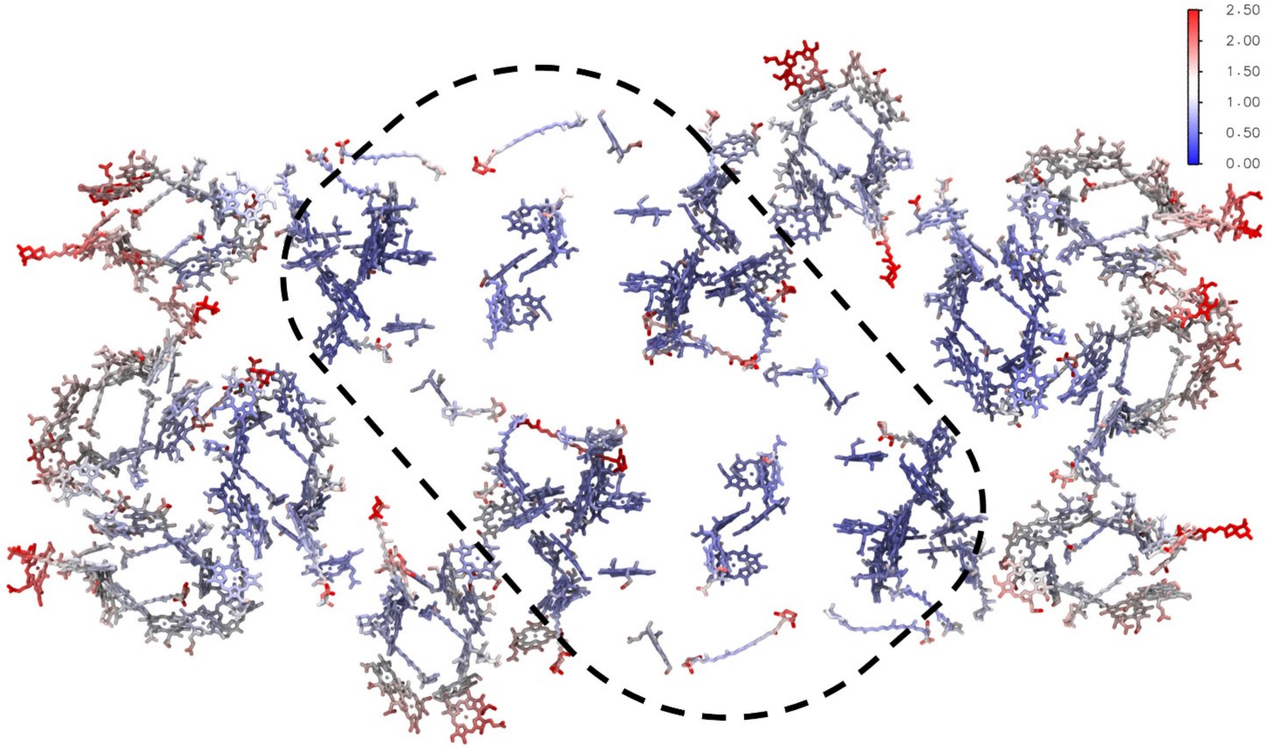

Fig. S4. Fluctuations of pigment molecules. The pigment molecules are viewed from the stroma side. The color scale bar shows the RMSF values. Red represents a high RMSF value, and blue represents a small RMSF value.

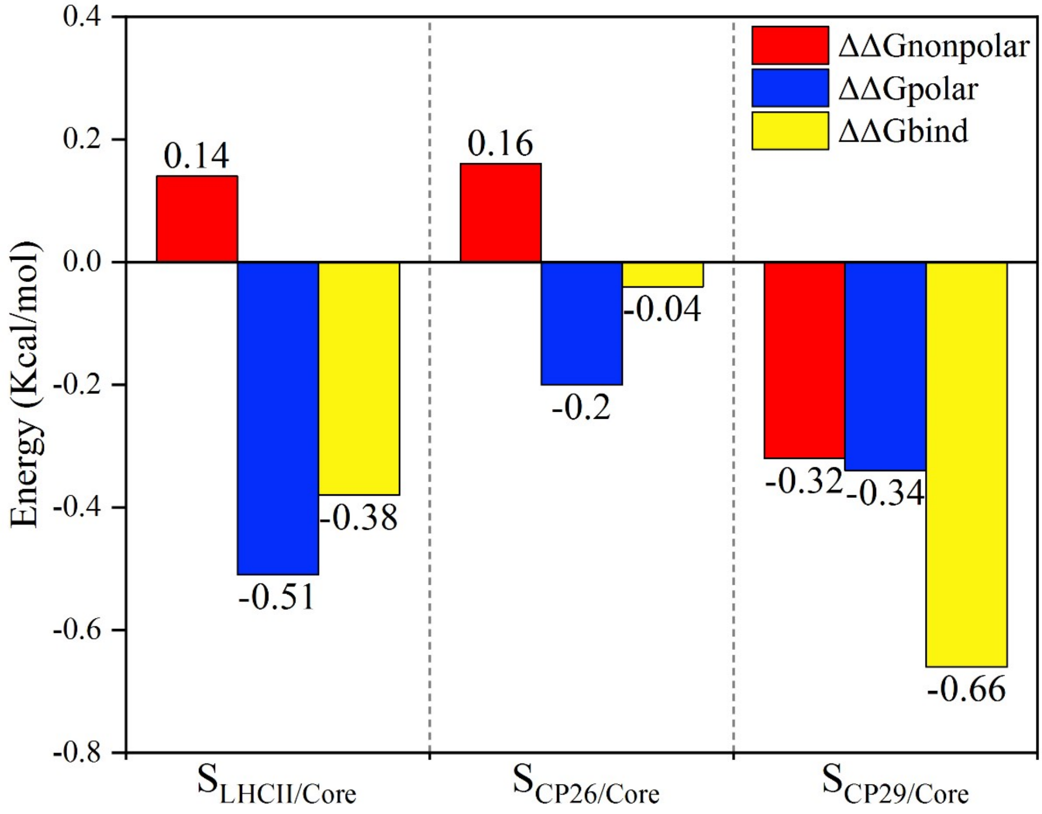

Fig. S5. The difference between the average binding free energy of HB/SB-involved hot spots and that of all hot spots. Results were obtained by subtracting all hotspot's average binding free energy from the average binding free energy of hot spots involved in hydrogen bonding.

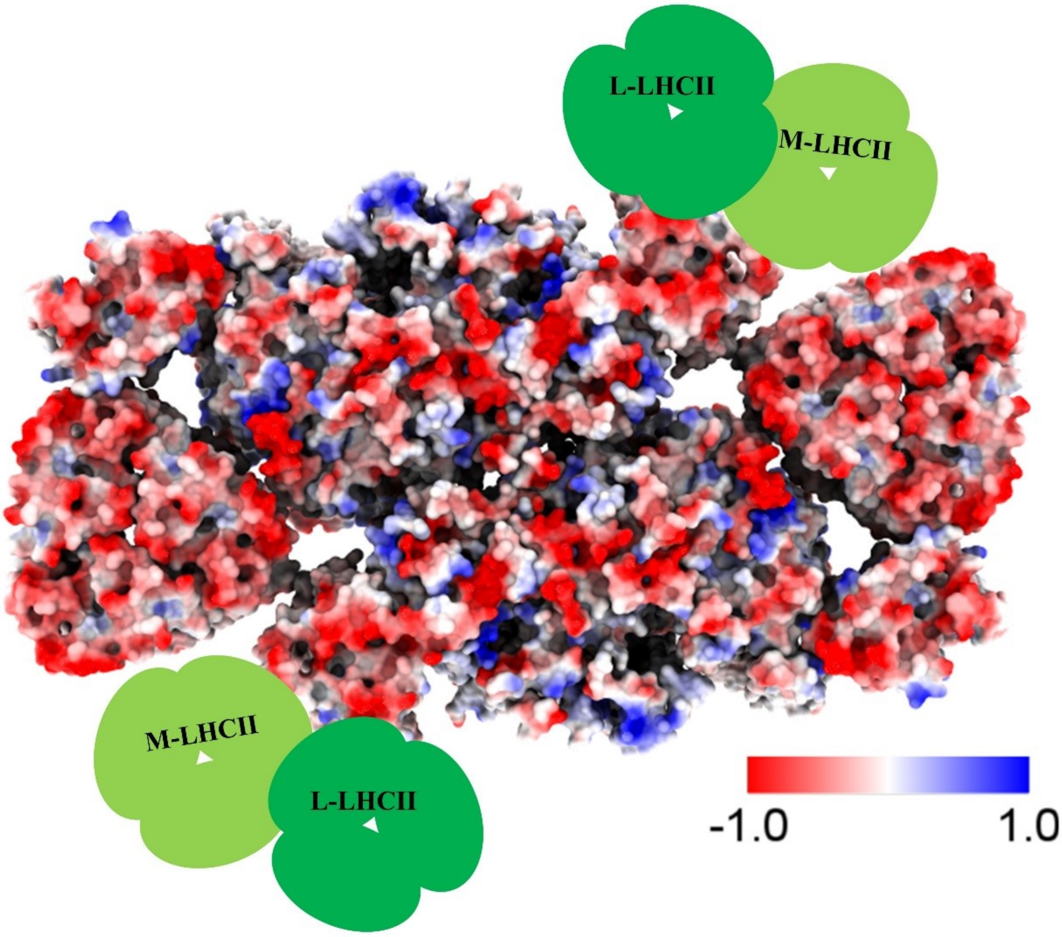

Fig. S6. Protein surface electrostatic potential of PSII-LHCII complex, viewed from the stromal side. The protein surface is colored based on the electrostatic potentials (kT/e), where the residues with positive potential are in blue, and those with negative potentials are in red. Dark green and light green represent potential locations of L-LHCII and M-LHCII, respectively (Sheng et al., 2019).

Table S1. Abbreviations and full names of ten cofactors

Abbreviation	Full name
PHO	pheophytin
BCR	β-carotene
PL9	plastoquinone-9
LHG	1,2-dipalmitoyl-phosphatidyl-glycerole
SQD	sulfoquinovosyldiacylglycerol
LMG	1,2-distearoyl-monogalactosyl-diglyceride
DGD	digalactosyl diacyl glycerol
LUT	lutein
XAT	violaxanthin
NEX	neoxanthin

Table S2. Different internal dielectric constant values influence the binding free energy calculation results of the implicit membrane model and the implicit solvent model, taking $S_{LHCII/core}$ as an example.

Methods	Indi[a]	ΔG_{vdW}	ΔG_{np}	ΔG_{elec}	ΔG_{PB}	$\Delta G_{nonpolar}$[b]	ΔG_{polar}[c]	ΔG_{bind}[d]
Implicit membrane model	10	-100.8±13.6	-11.3±1.0	92.1±3.2	0.0	-112.1	92.1	-20.0±15.4
	15	-100.8±13.6	-11.3±1.0	71.6±2.7	0.0	-112.1	71.6	-40.5±15.5
	20	-100.8±13.6	-11.3±1.0	59.2±2.5	0.0	-112.1	59.2	-52.9±15.5
	25	-100.8±13.6	-11.3±1.0	50.7±2.3	0.0	-112.1	50.7	-61.4±15.5
	30	-100.8±13.6	-11.3±1.0	44.5±2.1	0.0	-112.1	44.5	-67.6±15.5
Implicit solvent model	1	-100.8±13.6	-11.3±1.0	716.5±55.0	-665.8±49.4	-112.1	50.7	-61.4±20.0
	2	-100.8±13.6	-11.3±1.0	358.3±27.5	-327.1±24.4	-112.1	31.2	-80.9±16.8
	3	-100.8±13.6	-11.3±1.0	238.8±18.3	-214.9±16.0	-112.1	24.0	-88.1±16.2
	4	-100.8±13.6	-11.3±1.0	179.1±13.8	-159.0±11.7	-112.1	20.2	-91.9±15.9
	5	-100.8±13.6	-11.3±1.0	143.3±11.0	-125.3±9.2	-112.1	18.0	-94.1±15.8
	6	-100.8±13.6	-11.3±1.0	119.4±9.2	-102.9±7.5	-112.1	16.6	-95.5±15.7
	7	-100.8±13.6	-11.3±1.0	102.4±7.9	-86.8±6.3	-112.1	15.5	-96.6±15.6
	8	-100.8±13.6	-11.3±1.0	89.6±6.9	-74.8±5.4	-112.1	14.8	-97.3±15.5

[a]Internal dielectric constant

[b]formula (6), [c]formula (7), [d]formula (5)

Table S3. Binding free energy of different antenna-core interfaces was calculated using the implicit water model (kcal/mol). ΔG_{vdW}, the van der Waals interaction energy; ΔG_{np}, the non-polar solvation energy; ΔG_{elec}, the electrostatic interaction energy; ΔG_{PB}, the electrostatic solvation energy; $\Delta G_{nonpolar}$, the contribution of hydrophobic interactions; ΔG_{polar}, the gift of electrostatic interactions; ΔG_{bind}, the binding free energy.

Methods	Interface	ΔG_{vdW}	ΔG_{np}	ΔG_{elec}	ΔG_{PB}	$\Delta G_{nonpolar}$[a]	ΔG_{polar}[b]	ΔG_{bind}[c]
Implicit Solvent Model	$S_{LHCII/core}$	-103.5±12.5	-11.4±1.0	718.6±51.2	-668.2±45.0	-114.9	50.4	-64.5±18.0
	$S_{CP26/core}$	-46.5±5.3	-6.2±0.5	212.1±43.9	-186.4±43.4	-52.7	25.7	-27.0±8.2
	$S_{CP29/core}$	-167.4±8.5	-18.5±1.4	531.3±71.7	-462.9±65.5	-185.9	68.4	-117.5±15.5

[a]formula (6), [b]formula (7), [c]formula (5)

Table S4. Binding free energy of $S_{LHCII/CP26}$ and $S_{LHCII/CP29}$ (kcal/mol).

Interface	ΔG_{vdW}	ΔG_{np}	ΔG_{elec}	ΔG_{PB}	$\Delta G_{nonpolar}$[a]	ΔG_{polar}[b]	ΔG_{bind}[c]
$S_{LHCII/CP26}$	-36.6±6.4	-4.6±0.8	46.0±1.8	0	-41.2	46.0	4.8±7.0
$S_{LHCII/CP29}$	-5.8±0.9	-0.7±0.1	48.1±2.1	0	-6.5	48.1	41.5±2.0

[a]formula (6), [b]formula (7), [c]formula (5)

Table S5. Binding free energy and corresponding component contribution of hot spots on $S_{LHCII/Core}$ and $S_{CP26/Core}$.

System	Residue	ΔG_{vdW}	ΔG_{elec}	ΔG_{PB}	$\Delta G_{nonpolar}{}^{a}$	$\Delta G_{polar}{}^{b}$	$\Delta G_{bind}{}^{c}$	Proteins
$S_{LHCII/Core}$	L204	-1.47	-3.14	2.94	-1.47	-0.20	-1.68	CP43
	S205	-1.31	-5.97	4.91	-1.31	-1.06	-2.37	CP43*
	P206	-2.99	-1.18	1.88	-2.99	0.70	-2.29	CP43
	F210	-2.82	-0.96	2.05	-2.82	1.09	-1.73	CP43
	L96	-1.46	-1.22	1.08	-1.46	-0.13	-1.59	PsbW
	N103	-5.03	-9.56	12.07	-5.03	2.51	-2.52	PsbW*
	W107	-3.50	-0.98	2.49	-3.50	1.51	-2.00	PsbW*
	F110	-2.58	-0.24	1.07	-2.58	0.83	-1.75	PsbW
	S125	-1.44	-7.33	6.04	-1.44	-1.28	-2.72	PsbW*
	G126	-0.67	-4.07	3.12	-0.67	-0.95	-1.62	PsbW*
	L127	-2.30	-1.10	1.53	-2.30	0.43	-1.87	PsbW
	F81	-2.66	-0.49	1.57	-2.66	1.08	-1.58	LHCII
	L85	-4.21	1.23	0.15	-4.21	1.38	-2.83	LHCII
	G89	-1.76	-0.42	0.45	-1.76	0.03	-1.72	LHCII
	V90	-1.58	-3.83	2.37	-1.58	-1.46	-3.04	LHCII*
	L113	-2.65	-4.27	3.62	-2.65	-0.65	-3.30	LHCII*
	L164	-1.95	0.39	-0.19	-1.95	0.21	-1.75	LHCII
	L166	-1.88	0.70	-0.43	-1.88	0.28	-1.60	LHCII
	L213	-4.63	-0.27	2.38	-4.63	2.10	-2.53	LHCII
	P216	-1.92	-1.62	1.80	-1.92	0.19	-1.74	LHCII
	E175	-0.98	44.74	-41.09	-0.98	3.64	2.66	LHCII*
	K179	-0.88	-53.75	57.39	-0.88	3.64	2.76	LHCII
	D215	-1.82	51.50	-47.08	-1.82	4.42	2.60	LHCII
$S_{CP26/Core}$	F182	-3.31	-3.46	4.33	-3.31	0.87	-2.44	CP43
	W33	-1.29	-4.82	4.48	-1.29	-0.34	-1.63	PsbZ*
	L39	-2.72	-5.95	6.20	-2.72	0.25	-2.47	CP26*
	L231	-2.63	0.37	-0.07	-2.63	0.3	-2.33	CP26*
	I234	-1.91	0.93	-0.67	-1.91	0.26	-1.65	CP26

[a]formula (6), [b]formula (7), [c]formula (5)

*Residues involved in hydrogen bonds/salt bridges formation

Table S6. Binding free energy and corresponding component contribution of hot spots on $S_{CP29/Core}$.

Residue	ΔG_{vdW}	ΔG_{elec}	ΔG_{PB}	$\Delta G_{nonpolar}$[a]	ΔG_{polar}[b]	ΔG_{bind}[c]	Proteins
T228	-3.11	-3.21	3.76	-3.11	0.55	-2.56	D1
E229	-3.01	59.33	-57.91	-3.01	1.42	-1.59	D1*
N230	-1.74	-8.21	5.82	-1.74	-2.39	-4.13	D1*
R127	-1.48	-92.65	91.19	-1.48	-1.46	-2.94	CP47*
R220	-1.00	-93.11	92.48	-1.00	-0.63	-1.63	CP47
R230	-1.42	-98.35	94.85	-1.42	-3.50	-4.92	CP47*
D125	-0.47	77.00	-74.03	-0.47	2.97	2.5	CP47
P25	-3.41	2.36	-1.00	-3.41	1.35	-2.06	PsbH
L26	-1.80	0.77	-0.71	-1.80	0.06	-1.73	PsbH
Y30	-4.35	-5.69	6.71	-4.35	1.02	-3.32	PsbH*
G31	-1.88	-2.60	2.86	-1.88	0.26	-1.62	PsbH*
K32	-3.94	-120.65	120.77	-3.94	0.12	-3.82	PsbH*
V33	-3.02	-2.58	2.22	-3.02	-0.36	-3.38	PsbH*
P35	-2.10	-1.16	1.35	-2.10	0.19	-1.91	PsbH
E29	-1.53	83.26	-78.91	-1.53	4.34	2.82	PsbH
Q9	-0.51	-5.05	3.37	-0.51	-1.68	-2.19	PsbL*
Y45	-3.21	-0.70	1.44	-3.21	0.74	-2.47	CP29
L46	-3.37	-5.21	4.44	-3.37	-0.76	-4.14	CP29*
Q47	-4.03	-6.54	6.63	-4.03	0.10	-3.93	CP29*
Y48	-6.15	-1.54	5.03	-6.15	3.50	-2.65	CP29*
Y50	-5.87	-3.79	6.28	-5.87	2.49	-3.38	CP29*
L53	-4.59	-3.01	3.56	-4.59	0.55	-4.04	CP29*
Q55	-7.72	-10.11	13.68	-7.72	3.57	-4.15	CP29*
N56	-4.58	-13.52	11.01	-4.58	-2.51	-7.09	CP29*
K59	-2.78	-106.82	106.78	-2.78	-0.04	-2.83	CP29*
N60	-3.04	-5.87	6.54	-3.04	0.67	-2.37	CP29*
L80	-3.39	-0.80	1.93	-3.39	1.13	-2.26	CP29
S84	-0.57	-10.46	8.60	-0.57	-1.86	-2.43	CP29*
D49	-1.03	19.00	-16.44	-1.03	2.56	1.54	CP29*

[a]formula (6), [b]formula (7), [c]formula (5)

*Residues involved in hydrogen bonds/salt bridges formation

Table S7. Hydrogen bonds and salt bridges at the antenna-core interfaces in the cryo-EM structure.

	Hydrogen bond		Salt bridge		Distance (Å)[a]	Subunits in PSII core
	Donor	Acceptor	Acidic	Basic		
$S_{LHCII/core}$	ASN_88@Nδ	ASN_103@Oδ	-	-	3.20	PsbW
	TRP_107@Nε	ASN_88@Nδ	-	-	3.44	PsbW
	TRP_107@Nε	ASN_88@Oδ	-	-	2.89	PsbW
$S_{CP26/core}$	SER_59@Oγ	LEU_231@O	-	-	2.75	PsbZ
	LYS_37@Nζ	LEU_39@O	-	-	3.18	PsbZ
$S_{CP29/core}$	GLN_55@Nε	THR_10@O	-	-	2.64	CP47
	GLN_47@Nε	TYR_30@O	-	-	2.77	PsbH
	TYR_50@Oη	ASP_125@Oδ	-	-	2.82	CP47
	ASN_60@Nδ	GLU_229@Oε	-	-	2.84	D1
	ARG_69@Nη	ARG_127@Nη	-	-	3.12	CP47
	GLN_81@Nε	GLU_29@Oε	-	-	3.18	PsbH
	TYR_48@N	GLY_31@O			3.32	PsbH
	ASN_14@Nδ	ASP_54@N	-	-	3.36	CP47
	GLN_47@Nε	LYS_32@N	-	-	3.46	PsbH
	VAL_33@N	TYR_45@O			3.04	PsbH
	ASN_230@N	GLN_55@O			3.44	D1
	ARG_476@Nη	LEU_53@O	-	-	3.50	CP47
	-	-	ASP49	LYS130	3.91	CP47
	-	-	GLU85	LYS32	5.52	PsbH
	-	-	ASP477	LYS59	5.85	CP47
	-	-	ASP483	LYS59	4.89	CP47

[a]Hydrogen bond based on a distance between non-hydrogen atoms of less than 3.5 Å. Salt bridge based on a distance between acidic oxygen atoms and basic nitrogen atoms of less than 6 Å.

Supplementary Video 1:

Supplementary References

Sheng X, Watanabe A, Li AJ, Kim E, Song CH, et al. (2019) Structural insight into light harvesting for photosystem II in green algae. Nature Plants **5:** 1320-1330
